# Supplementary material for: Fortification of Cereal-Based Food with Lactobacillus rhamnosus GG and Bacillus coagulans GBI-30 and Their Survival During Processing
Source: Foods. 2025 Jun 25;14(13):2250. doi: 10.3390/foods14132250 (PMC12248632; doi:10.3390/foods14132250)
Supplement: Supplementary file 1 [file foods-14-02250-s001.zip › foods-3661403-supplementary.pdf]

Supplementary Material

Table S1. Viable cell counts (log CFU/g) of LGG and BC30 in the blended flour used for pasta and noodle preparation

Sample	Location	Replicates	Mean	SD
	Top	9.07, 9.04, 9.21		
**Pasta Flour – LGG**	Middle	9.25, 9.09, 9.31	9.1389	0.0872
	Bottom	9.28, 9.12, 9.06		
	Top	9.22, 9.14, 9.17		
**Pasta Flour – BC30**	Middle	9.25, 9.18, 9.22	9.1967	0.0676
	Bottom	9.33, 9.16, 9.10		
	Top	9.62, 9.58, 9.61		
**Noodle Flour – LGG**	Middle	9.59, 9.65, 9.57	9.5956	0.0544
	Bottom	9.68, 9.57, 9.49		
	Top	9.70, 9.68, 9.71		
**Noodle Flour – BC30**	Middle	9.65, 9.69, 9.68	9.6944	0.0270
	Bottom	9.75, 9.69, 9.70		
